# Combined effects of age and hearing impairment on utterances and requests for clarification in spontaneous conversation and a referential communication task

**DOI:** 10.1111/1460-6984.12940

**Published:** 2023-08-17

**Authors:** Rachel J. Ellis, Jerker Rönnberg, Charlotta Plejert

**Affiliations:** ^1^ Department of Behavioural Sciences and Learning Linköping University Linköping Sweden; ^2^ Department of Biomedical and Clinical Sciences Linköping University Linköping Sweden

**Keywords:** adults, hearing impairment, social interaction, speech

## Abstract

**Background:**

The impact of hearing impairment is typically studied in terms of its effects on speech perception, yet this fails to account for the interactive nature of communication. Recently, there has been a move towards studying the effects of age‐related hearing impairment on interaction, often using referential communication tasks; however, little is known about how interaction in these tasks compares to everyday communication.

**Aims:**

To investigate utterances and requests for clarification used in one‐to‐one conversations between older adults with hearing impairment and younger adults without hearing impairment, and between two younger adults without hearing impairment.

**Methods & Procedures:**

A total of 42 participants were recruited to the study and split into 21 pairs, 10 with two younger adults without hearing impairment and 11 with one younger adult without hearing impairment and one older participant with age‐related hearing impairment (hard of hearing). Results from three tasks—spontaneous conversation and two trials of a referential communication task—were compared. A total of 5 min of interaction in each of the three tasks was transcribed, and the frequency of requests for clarification, mean length of utterance and total utterances were calculated for individual participants and pairs.

**Outcomes & Results:**

When engaging in spontaneous conversation, participants made fewer requests for clarification than in the referential communication, regardless of hearing status/age (*p* ≤ 0.012). Participants who were hard of hearing made significantly more requests for clarification than their partners without hearing impairment in only the second trial of the referential communication task (*U* = 25, *p* = 0.019). Mean length of utterance was longer in spontaneous conversation than in the referential communication task in the pairs without hearing impairment (*p* ≤ 0.021), but not in the pairs including a person who was hard of hearing. However, participants who were hard of hearing used significantly longer utterances than their partners without hearing impairment in the spontaneous conversation (*U* = 8, *p* < 0.001) but not in the referential communication tasks.

**Conclusions & Implications:**

The findings suggest that patterns of interaction observed in referential communication tasks differ to those observed in spontaneous conversation. The results also suggest that fatigue may be an important consideration when planning studies of interaction that use multiple conditions of a communication task, particularly when participants are older or hard of hearing.

**WHAT THIS PAPER ADDS:**

**What is already known on this subject**

Age‐related hearing impairment is known to affect communication; however, the majority of studies have focused on its impact on speech perception in controlled conditions. This indicates little about the impact on everyday, interactive, communication.

**What this study adds to the existing knowledge**

We investigated utterance length and requests for clarification in one‐to‐one conversations between pairs consisting of one older adult who is hard of hearing and one younger adult without hearing impairment, or two younger adults without hearing impairment. Results from three tasks (two trials of a referential communication task and spontaneous conversation) were compared. The findings demonstrated a significant effect of task type on requests for clarification in both groups. Furthermore, in spontaneous conversation, older adults who were hard of hearing used significantly longer utterances than their partners without hearing impairment. This pattern was not observed in the referential communication task.

**What are the potential or actual clinical implications of this work?**

These findings have important implications for generalizing results from controlled communication tasks to more everyday conversation. Specifically, they suggest that the previously observed strategy of monopolizing conversation, possibly as an attempt to control it, may be more frequently used by older adults who are hard of hearing in natural conversation than in a more contrived communication task.

## INTRODUCTION

Hearing impairment is one of the main causes of disability in older adulthood and is associated with significant negative psychosocial effects, leading to reduced quality of life and poorer psychological and physical health (Gopinath et al., 2012). A recent report by the World Health Organisation (WHO) (2021) projects that by 2050 almost 2.5 billion people will be living with hearing impairment, with 700 million of these having hearing impairment severe enough to require treatment (rising from 430 million today). Hearing impairment is particularly prevalent in older adults, with two‐thirds of those over the age of 70 having a hearing impairment severe enough to affect communication (Lin et al., 2011).

The impact of hearing impairment is often studied in terms of its effects on speech recognition (e.g., Davidson et al., 2021; Humes, 2021; Rönnberg et al., 2016); however, communication is an interactive process that requires the ability to perceive, produce and appropriately react to information. Thus, the tendency for studies on the effects of hearing impairment to focus primarily on its influence on speech recognition may mean that they fail to capture the true impact of hearing impairment on interpersonal communication. As such, there is a need for studies that focus not only on the person who is hard of hearing, but also on their conversational partner and the collaborative nature of their interaction.

Findings relating to the effects of hearing impairment on interaction suggest that individuals who are hard of hearing display different patterns of communication to those without hearing impairment. Individuals who are hard of hearing tend, for example, to demonstrate a higher incidence of conversational breakdown and repair (Pajo, 2013), and to use different repair strategies than those without hearing impairment (Lind, Hickson and Erber, 2006), and are more likely than those without hearing impairment to replace full conversational turns with fillers and backchannels (e.g., ‘hmm’). Individuals who are hard of hearing also show an increased tendency either to avoid conversation altogether (Stephens et al., 1999) or to use more monologues and interruptions (Tye‐Murray and Witt, 1996) in order to minimize the possibility of conversational breakdown.

In cases where breakdown occurs, one way to try to repair this is to make a clarification request. Requests can be specific (e.g., ‘what colour did you say the door was?’) or non‐specific, (e.g., ‘what?’), sometimes known as an open‐class repair initiator (Drew, 1997). Caissie and Gibson (1997) found that adults who are hard of hearing are more likely to use non‐specific than specific requests for clarification when talking to unfamiliar partners in an environment with background noise. However, they argue that to use a specific request for confirmation requires the individual to have heard much of what was said to begin with, meaning they can only be used in cases where difficulty is minimal, which may, at least in part, explain their relatively higher success rate. Ibertsson et al. (2009) investigated requests for clarification between teenagers with a cochlear implant and peers without hearing impairment in a referential communication task. Results showed that participants with cochlear implants made significantly more requests for clarification than those without hearing impairment, and the types of clarification requests used differed between the two groups, although requests for clarification of new information were the most frequently used in both. Furthermore, they emphasized the importance of focusing not only on the speech of the individual who is hard of hearing, but also on the conversation as a collaborative achievement. This view was echoed by Görsdorf (2012), who argued that interactional studies of hearing impairment may provide relief and empowerment to individuals who are hard of hearing due to the recognition that the burden of responsibility for successful communication is on all participants in the conversation, not just those who are hard of hearing (Görsdorf, 2012). Like Ibertsson et al. (2009), Pajo (2013) also found, in a study of adults (aged 43–69 years) who were hard of hearing and familiar partners without hearing impairment, that individuals who were hard of hearing made more requests for clarification than their partners, yet did not differ in the types of clarification request used. However, Laakso et al. (2019) investigated non‐specific requests for clarification in middle‐aged adults with mild to moderate acquired hearing impairment and interlocutors without hearing impairment, finding no difference in frequency of their use between the two groups.

Most studies of the effects of hearing impairment on interaction have based their analyses on conversations in uncontrolled environments, often in everyday life (e.g., Laakso et al., 2019; Pajo, 2013). While this is beneficial in terms of external validity, it would be time‐consuming and difficult to use these techniques to compare the effects of different listening conditions (e.g., aided and unaided, different levels and type of background noise or hearing aid signal processing methods) on interaction, as opposed to on speech recognition, as is typically the case. One way this could be investigated would be via using repeated trials of tasks designed to elicit dialogue in a controlled environment. While a number of studies have used such tasks, there has been limited investigation into the effects of repeated trials, or on how interaction in such tasks compares with natural conversation, thus affecting how generalizable the results of such studies would be. Two exceptions are the studies conducted by Wilson et al. (1998) and Ibertsson et al. (2009).

Wilson et al. (1998) observed more breakdowns when an older adult with mild‐to‐moderate hearing impairment engaged in natural conversation with an investigator than when the investigator used breakdown elicitation techniques (e.g., obscuring mouth with hand, rapid speech rate). However, they observed no difference between the frequency of breakdowns in the natural conversation with the investigator and group conversation with three other individuals who were hard of hearing. In terms of types of repair (of which clarification requests are an example), similar strategies were used in both the individual tasks, but differed in the group task. Ibertsson et al. (2009) used two repetitions of a referential communication task in their study, and found that higher numbers of words were used by both interlocutors in the second task compared with the first. However, in their study participants were assigned roles as either a describer or a receiver in the referential communication task, and these roles were switched in the two trials. This suggests that it is important to consider task type and partner characteristics when interpreting and generalizing findings.

To summarize, the relative lack of studies on the impact of hearing impairment on interaction, along with the heterogeneity of participants (in terms of age, severity and aetiology of hearing loss, etc.) and differences in the methodologies employed mean it is difficult to know the extent to which research findings can be generalized. As such, this study will investigate the combined effects of age and age‐related hearing impairment on interaction in spontaneous conversation and a referential communication task. Specifically, we will investigate the following research questions:

- Do mean length of utterance, number of utterances, and/or frequency and type of requests for clarification differ:
  ○ At a pair level between pairs consisting of one older adult who is hard of hearing and one younger adult without hearing impairment, and pairs consisting of two younger adults without hearing impairment?
  ○ At an individual level between older adults who are hard of hearing and younger adults without hearing impairment?
- How are the measures detailed above affected by task type (two trials of a referential communication task and spontaneous conversation) when interlocutors are not assigned specific roles?

## METHODS

### Participants

A total of 42 (19 female, 23 male) participants were recruited to the study and split into 21 pairs. Ten of the pairs consisted of two young participants without hearing impairment, while the other 11 pairs consisted of one young participant without hearing impairment and one older participant who was hard of hearing. All participants were fluent in Swedish and reported having normal or corrected to normal vision and no neurological conditions. Of the 11 older participants, all had hearing impairment (which was checked using pure tone audiometry at the test session), and reported not having, or not using, hearing aids in everyday life. Participants in the older group were required to be 60 or older (median = 67, range = 62–84 years old). In the younger group, participants were required to be 35 or younger (median = 24, range = 19–31 years old) and to not have a hearing impairment (based on self‐report here). Participants were not known to their partners before testing; however, participants in one of the young pairs turned out to be acquaintances. Participants were recruited via posters and existing connections with the research group and received monetary compensation (approximately US$30) for participating. Ethics approval was granted by the Ethical Review Board in Linköping, Sweden (ref. number 2015/197‐31).

### Communication tasks

#### Referential communication

The referential communication task was adapted from the Diapix UK (Baker and Hazan, 2011), which was in turn adapted from the Diapix (Van‐Engen et al., 2010). The Diapix is a dialogue‐elicitation task completed in pairs. The task requires two people to work together to verbally complete a ‘spot‐the‐difference’‐type task in which each talker can only see one of the two images that are to be compared. Participants were told that their task was to discuss the pictures, which showed illustrated outdoor scenes, with the aim of identifying differences between them. Four pairs of pictures were adapted from the Diapix UK materials. Adaptations involved translating text (in the form of signs and speech bubbles) into Swedish and editing the materials to leave 10 differences in each pair of pictures. This was done to limit the time needed to complete the task, as each participant pair was given three pairs of Diapix pictures to solve. Translations were performed by the first author, a native English speaker proficient in Swedish, and approved by native Swedish speakers proficient in English. Participants were not told how many differences there were between the pictures, as pilot testing revealed that this had a noticeable effect on interaction, whereby discussion was limited to listing and counting differences, rather than on discussing the pictures in detail. The order in which the three pairs of Diapix pictures were administered was counterbalanced, including one practice round which was completed with the tester in the room. The tester left the room for the remaining two Diapix tasks, returning only to swap the pictures when participants indicated (by fetching the tester, who sat outside the room) that they were finished with the first task. Participants sat opposite each other at a table. The Diapix pictures were attached to boards and positioned in front of participants and angled to one side so as to allow participants to see each other.

#### Spontaneous communication

Participants were left alone in the test room for 5 min while the tester left to collect materials. The interaction between participants during this time formed the spontaneous communication condition.

### Procedure

**FIGURE 1:**
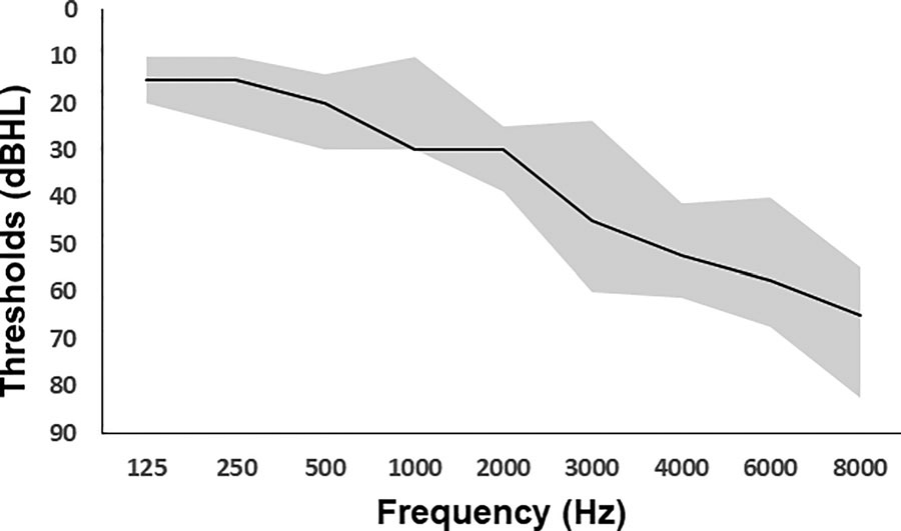
Median hearing thresholds for participants with hearing impairment. Note: The shaded area indicates the interquartile range. Thresholds up to 80 dBHL were measured; any threshold greater than that was assigned a value of 90 dBHL

Testing took place in a quiet room at Linköping University. Participants in the older group were aware that the aim of the study was to look at the effects of hearing impairment on interaction, and were explicitly asked not to mention their hearing impairment to their partner. All testing was conducted unaided. However, six of the participants in this group wore a dummy hearing aid that was not turned on during testing, as an original aim of the study was also to look at the role of hearing aid visibility on interaction. This turned out not to be possible, as many of the participants who were hard of hearing explicitly mentioned this to their partners during testing, thus creating a confound with the hearing aid visibility manipulation. In the cases where a dummy hearing aid was used, this was provided at the start of the session, and was fit with a flexible open ear mould so as not to occlude the ear canal. These preparations took place before the younger participants arrived. Pure tone audiometry was conducted (for the older participants only) at the end of the test session (see Figure 1 for median air conduction pure tone thresholds). Pure tone thresholds were measured at 125, 250, 500, 1000, 2000, 4000, 6000 and 8000 Hz in both ears. Thresholds were measured up to 80 dB HL—if a response was not obtained at that level, a value of 90 dB HL was used when calculating median thresholds for the group. Younger participants were told that the purpose of the study was to look how participants performed in a communication task, and were not made aware of the full focus of the study until the end of the test session.

All participants were first asked to confirm that they had read the information sheet that they had been sent previously, and were offered the opportunity to read it again. It was explained that they had the right to withdraw from the study, or to request a break, at any time, and they were asked to confirm that they consented to the session being video recorded. The tester then outlined what would happen during the session and offered participants refreshments before leaving the room to collect the materials for the referential communication task. The tester left the room for at least 5 min, and the interaction that took place between participants during this time was used for the spontaneous conversation condition. The tester then returned and the referential communication test was administered.

### Transcription and analysis

The first 5 min of each task (spontaneous conversation, Diapix 1 and Diapix 2) were transcribed based on video recordings and accompanying sound files, with any instances of unclear speech being discussed by at least two researchers. For one pair (pair 10), the final 5 min of the Diapix tasks was used instead of the first 5 min, as this pair began by each describing their pictures in detail to one another, and only began interaction after these monologues. In two cases (pairs 3 and 11), either the spontaneous conversation (pair 11) or the Diapix 2 (pair 3) had a total duration of less than 5 min (3 min 10 s and 4 min, respectively). In order to allow for more meaningful comparison between tasks, in these two cases, analysis of data from the other two tasks was also limited to 3 min 10 s or 4 min (rather than five) of interaction.

Analyses were focused on verbal interaction, but non‐verbal cues were used to aid interpretation where appropriate. The total number of utterances and the mean length of utterance were calculated. An utterance was defined as a turn, or partial turn if the speaker was interrupted by their interlocutor. The number of utterances (including fillers such as ‘err’, ‘um’, etc.) in each 5‐min sample were totalled and mean length of utterance, based on the total utterances in each sample, was calculated. Requests for clarification were then identified and coded for further analysis. A request for clarification was defined as being any request relating to a topic previously introduced by the conversational partner. Requests for clarification were coded according to the criteria used by Ibertsson et al. (2009), which was based on Caissie and Rockwell (1993), Tye‐Murray et al. (1995) and Caissie and Gibson (1997). Using this framework, requests for clarification were coded as falling into one of seven categories: non‐specific requests for clarification (e.g., ‘what?’), requests for repetition (e.g., ‘what did you say he was holding?’), requests for confirmation of new information (e.g., ‘are the flowers red?’), requests for confirmation of already given information (e.g., ‘did you say that the flowers were red?’), requests for elaboration (e.g., ‘what colour are the flowers?’), forced choice questions (e.g., ‘are the flowers white or red?’) requesting either confirmation or elaboration, and control questions to check that the partner understands (e.g., do you know what I mean?).

We also derived an extra category containing requests that were likely to be hearing related. This category included non‐specific requests for clarification, requests for repetition and requests for confirmation of already‐given information that related to uncertainty about what was said, rather than what was meant. Coding of clarification requests was conducted by one researcher and checked by a second, using the next‐turn‐proof procedure (Hutchby & Wooffitt, 1998: 15), in which the way an utterance immediately following a possible request was interpreted by an interlocutor aided its classification—cases that remained unclear were discussed until agreement was reached, or excluded in the few cases where poor intelligibility meant that classification was not possible.

### Statistical analyses

Statistical analyses were based on non‐parametric tests due to non‐normal distributions in the data. Friedman tests were used to examine the effect of task type on performance, with sign tests used for post‐hoc analyses to further examine the nature of the differences between the three task types. Post‐hoc tests were interpreted using the Bonferroni–Holm adjustment for multiple comparisons (Holm, 1979). Mann–Whitney *U*‐tests (based on mean ranks) were used to examine differences between groups. The alpha level was set at *p* < 0.05 and two‐tailed values are reported. Analyses comparing interaction including participants who were hard of hearing to interactions between two participants without hearing impairment were conducted at the pair level. In addition, interactions including a participant without hearing impairment and a participant who was hard of hearing were analysed at an individual level in order to investigate the effects of hearing impairment/age. All statistical analyses were conducted using IBM SPSS Statistics 25.

## RESULTS

**FIGURE 2:**
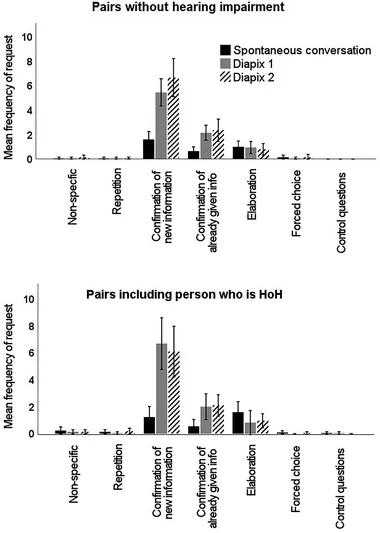
Mean frequency of the different types of clarification request for the pairs consisting of two younger adults without hearing impairment (upper) and the pairs consisting of one younger adult without hearing impairment and one older adult with hearing impairment (lower). Note: Black bars show data from the spontaneous conversation task; grey bars the Diapix 1; and patterned bars the Diapix 2. Error bars show the 95% confidence intervals

Descriptive statistics at the pair and individual levels are shown in Tables 1 and 2, respectively. Due to the low frequency of non‐specific requests for clarification, only frequencies of total requests for clarification and hearing‐related requests for clarification have been analysed further; however, the distribution of the different types of clarification request are shown in Figures 2 (pair data) and 3 (individual data).

**TABLE 1:** Descriptive statistics for the pairs consisting of two younger adults without hearing impairment, and of one younger adult without hearing impairment and one older adult with hearing impairment

		**Pairs without hearing impairment**		**Pairs including person who is hard of hearing**	
		**Median**	**Interquartile range**	**Median**	**Interquartile range**
Age (years)		24	23–27.5	–	–
Mean length of utterance	Spontaneous conversation	8.1	7.4–10.1	6.0	5.2–7.7
	Diapix 1	6.1	5.7–6.8	5.8	5.0–6.6
	Diapix 2	5.5	4.9–6.7	6.3	5.6–6.9
Total utterances	Spontaneous conversation	87.5	77.0–112.8	115.0	82.0–146.0
	Diapix 1	125.0	112.8–147.8	124.0	106.0–148.0
	Diapix 2	130.0	106.5–145.5	108.0	102.0–132.0
Total requests for clarification	Spontaneous conversation	7.0	4.5–8.3	7.0	5.0–11.0
	Diapix 1	17.0	14.0–20.5	19.0	16.0–25.0
	Diapix 2	20.0	15.5–26.3	20.0	15.0–23.0
Hearing‐related requests for clarification	Spontaneous conversation	0.5	0–1.3	1.0	0–2.0
	Diapix 1	1.0	0.75–2.0	1.0	0–2.0
	Diapix 2	2.0	1.0–2.5	1.0	0–3.0

**TABLE 2:** Descriptive statistics for the individual participants without hearing impairment and participants with hearing impairment in the pairs consisting of one younger adult without hearing impairment and one older adult with hearing impairment

		**Younger adults without hearing impairment**		**Older adults who are hard of hearing**	
		**Median**	**Interquartile range**	**Median**	**Interquartile range**
Age (years)		23.0	21.0–26.0	67.0	66.0–76.0
Mean length of utterance	Spontaneous conversation	3.9	3.4–5.5	8.4	6.7–10.3
	Diapix 1	5.9	4.8–7.0	5.6	4.8–6.2
	Diapix 2	6.0	4.8–7.0	6.3	4.7–7.3
Total utterances	Spontaneous conversation	56.0	40.0–73.0	59.0	42.0–75.0
	Diapix 1	63.0	52.0–73.0	61.0	54.0–73.0
	Diapix 2	56.0	52.0–67.0	52.0	48.0–65.0
Total requests for clarification	Spontaneous conversation	4.0	2.0–7.0	1.0	0–4.0
	Diapix 1	11.0	5.0–14.0	10.0	4.0–14.0
	Diapix 2	6.0	5.0–9.0	11.0	7.0–17.0
Hearing‐related requests for clarification	Spontaneous conversation	0	0–1.0	0	0–2.0
	Diapix 1	0	0–1.0	0	0–1.0
	Diapix 2	0	0–2.0	0	0–1.0

**FIGURE 3:**
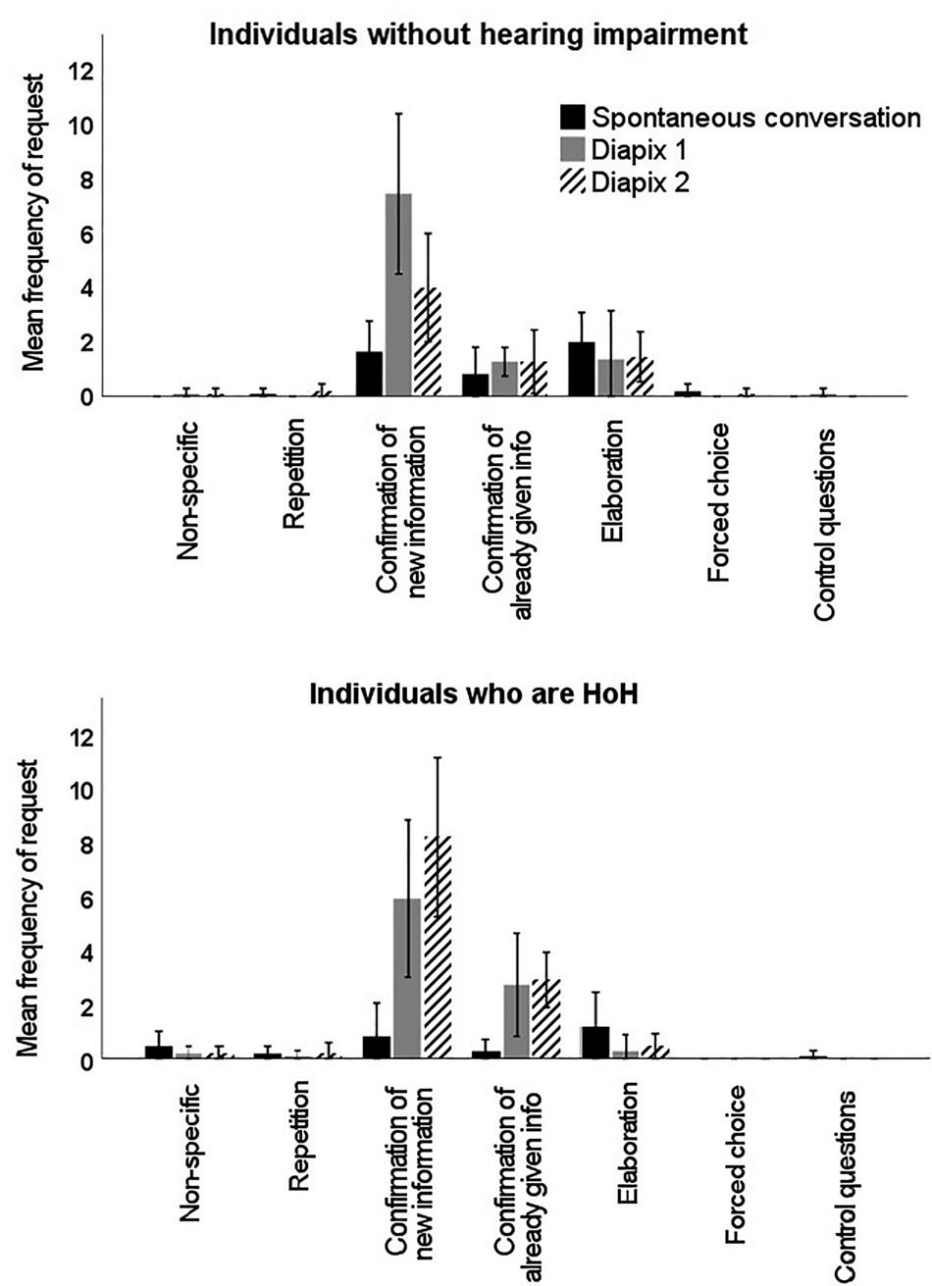
Mean frequency of the different types of clarification request for participants without hearing impairment (upper) and participants with hearing impairment (lower) in the pairs consisting of one younger adult without hearing impairment and one older adult with hearing impairment. Note: Black bars show data from the spontaneous conversation task; grey bars the Diapix 1; and patterned bars the Diapix 2. Error bars show the 95% confidence intervals

### Interaction between young adults without hearing impairment

See Table 3 for the results of the tests detailed below.

**TABLE 3:** Analyses (Friedman and sign tests) showing the effects of task type on outcomes at the pair level

	**Pairs without hearing impairment**	**Pairs including person who is hard of hearing**
Total requests for clarification	*χ* ^2^ = 16.80, *p* < 0.001	*χ* ^2^ = 13.82, *p* = 0.001
Spontaneous conversation × Diapix 1	*p* = 0.002	*p* = 0.001
Spontaneous conversation × Diapix 2	*p* = 0.002	*p* = 0.012
Diapix 1 × Diapix 2	*p* = 0.109	*p* = 1.00
Hearing‐related requests for clarification	*χ* ^2^ = 3.77, *p* = 0.152	*χ* ^2^ = 0.61, *p* = 0.970
Mean length of utterance	*χ* ^2^ = 12.60, *p* = 0.002	*χ* ^2^ = 1.64, *p* = 0.441
Spontaneous conversation × Diapix 1	*p* = 0.002	
Spontaneous conversation × Diapix 2	*p* = 0.021	
Diapix 1 × Diapix 2	*p* = 0.344	
Total utterances	*χ* ^2^ = 9.90, *p* = 0.007	*χ* ^2^ = 3.12, *p* = 0.211
Spontaneous conversation × Diapix 1	*p* = 0.021	
Spontaneous conversation × Diapix 2	*p* = 0.021	
Diapix 1 × Diapix 2	*p* = 1.00	

#### Clarification requests

There was a significant effect of task on total number of clarifications. Post‐hoc sign tests revealed significant differences between spontaneous conversation and both Diapix tasks, but not between the two Diapix tasks.

No effect of task on the total number of hearing‐related clarification requests was observed.

#### Mean length of utterance and number of utterances

A Friedman test revealed a significant effect of task on the mean length of utterance in the conversational pairs. Post‐hoc sign tests showed significant differences between spontaneous conversation and both the Diapix 1 and Diapix 2 tasks; however, mean length of utterance in the two Diapix tasks did not differ significantly.

A significant effect of task on total number of utterances was also observed. Post‐hoc sign tests again showed significant differences between spontaneous conversation and both the Diapix 1 and Diapix 2 tasks; however, there was no significant difference in total number of utterances in the two Diapix tasks.

### Interaction between older adults who were hard of hearing and young adults without hearing impairment

See Tables 3 (analyses at pair level) and 4 (analyses at the individual level) for the results of the tests detailed below.

**TABLE 4:** Analyses (Mann–Whitney *U*‐tests) showing the effects of age/hearing impairment on outcomes at the individual level in each of the three tasks for participants in the pairs consisting of one younger adult without hearing impairment and one older adult with hearing impairment

	**Spontaneous conversation**	**Diapix 1**	**Diapix 2**
Total requests for clarification	*U* = 34.5, *p* = 0.088	*U* = 54, *p* = 0.699	*U* = 25, *p* = 0.019
Hearing‐related requests for clarification	*U* = 50, *p* = 0.519	*U* = 57.5, *p* = 0.847	*U* = 56.5, *p* = 0.797
Mean length of utterance	*U* = 8, *p* < 0.001	*U* = 57, *p* = 0.847	*U* = 58, *p* = 0.898
Total utterances	*U* = 58, *p* = 0.898	*U* = 59, *p* = 0.949	*U* = 50.5, *p* = 0.519

#### Clarification requests

There was a significant effect of task on total number of clarifications at the pair level. Post‐hoc sign tests revealed significant differences between spontaneous conversation and both the Diapix 1 and Diapix 2 tasks, but not between the two Diapix tasks. No effect of task on the total number of hearing‐related clarification requests at the pair level was observed.

At the individual, rather than pair, level, Mann–Whitney *U*‐tests showed a significant effect of age/hearing impairment on the total number of clarifications in the Diapix 2 task, but not in spontaneous conversation or the Diapix 1 task. No significant effects of age/hearing impairment on the number of hearing‐related requests for clarification were observed in any of the tasks.

#### Mean length of utterance and number of utterances

There was no significant effect of task on either mean length of utterance or total number of utterances in the conversational pairs. Mann–Whitney *U*‐tests conducted to examine the effect of age/hearing impairment on outcomes at the individual, rather than pair, level revealed significant differences in mean length of utterance in spontaneous conversation but not in the Diapix 1 or Diapix 2 tasks. There was no effect of age/hearing impairment on total utterances in any of the tasks.

## DISCUSSION

The results of the current study demonstrated a significant effect of task type on requests for clarification in conversation between younger adults without hearing impairment, and between older adults who are hard of hearing and younger adults without hearing impairment. When engaging in spontaneous conversation, participants tended to make fewer requests for clarification than in the referential communication task, regardless of hearing status/age. At a pair level, there was no difference in the frequency of clarification requests between the first and second trial of the referential communication task, however, participants who were hard of hearing made significantly more requests for clarification than their partners without hearing impairment in the second trial only. This may indicate that the participants who were hard of hearing, who were also the older adults, were perhaps becoming fatigued at this point in the session, and were using clarification requests as a method of exercising control in the interaction, something that has been reported previously (Ibertsson et al., 2009).

Another method in which a speaker may attempt to increase control in interaction is by increasing utterance length. While mean length of utterance was longer in spontaneous conversation than in either of the Diapix trials in the pairs without hearing impairment, no such effect was observed in the pairs including a person who was hard of hearing. However, when the impact of age/hearing impairment was investigated, an imbalance in contribution was observed in the spontaneous conversation task, where participants who were hard of hearing used significantly longer utterances than their partners without hearing impairment. This pattern was not observed in the referential communication tasks. These findings are consistent with Caissie and Rockwell (1994) who based their analyses on naturally occurring conversation, yet not with Ibertsson et al. (2009) who used a dialogue elicitation task, suggesting that caution should be taken when generalizing findings to everyday communication. Methods of controlling conversation have been reported to be more commonly used when conversing with unfamiliar partners (Tye‐Murray et al., 1995), so it may be that different results would have been observed had participants in this study known each other beforehand.

In relation to the types of clarification requests used, only three types were used regularly—requests for confirmation of new information, requests for confirmation of already given information, and requests for elaboration. This is broadly consistent with the findings of Gibson and Caissie (1994) and Ibertsson et al. (2009), and an extension of that study reported by Sandgren et al. (2011), which showed that requests for confirmation were the most frequently used. In the referential communication task, requests for confirmation of new information were mostly commonly used, followed by requests for confirmation of already given information, then requests for elaboration, with the same pattern observed between pairs and between individuals without hearing impairment and those who were hard of hearing. In spontaneous conversation, each of these types of clarification requests were more evenly used, suggesting once more that caution is needed when generalizing findings from dialogue elicitation tasks. This pattern was again common between pairs and between individuals without hearing impairment and those who were hard of hearing. Non‐specific requests for clarification were rarely used in any of the tasks. This is in conflict with the findings reported by Caissie and Gibson (1997) who also looked at clarification requests in a spontaneous conversation between adults who had an acquired hearing impairment and unfamiliar partners without hearing impairment. However, in their study, the interaction took place in a background of noise, whereas in the present study, the task was conducted in a quiet room. Furthermore, studies based on analysing naturally occurring conversation in everyday life have shown that non‐specific requests for clarification are regularly used by people who are hard of hearing (Pajo, 2013; Laakso et al., 2019), so it is likely that their relative infrequency in the present study relates to the optimal conditions (one‐to‐one conversation in a quiet room) in which the testing took place.

Future research using different communication tasks and conducted in environments of varying complexity, for example with different types and levels of background noise, are needed to give a greater understanding of the impact of hearing impairment on interaction in different conditions, including everyday life. Furthermore, it may be of interest to investigate associations between cognition and different types of clarification request, both in relation to the complexity of the communication environment and the task being conducted, and to the ability to effectively use either requests for clarification or the information provided in response. Varying pair dynamics (on, for example, familiarity, hearing impairment, age, gender), along with including participants with differing degrees and types of hearing impairment and assistive technology use (see also the pilot study reported by McInerny & Walden, 2013) would be beneficial, as it is important to recognize the heterogeneity of this group. It may also be helpful to vary the order in which different tasks are given, as in this study the spontaneous conversation condition always took place before the referential communication task—this may mean that any possible effects relating to fatigue were more likely to impact the latter. Furthermore, instead of controlling for relative length of conversation between different tasks, it could be interesting to control for number of utterances between tasks. This is something that varied in some of our data, however, as this was a naturally occurring difference between the tasks, we felt controlling for it would lead us to underestimate some of the potential issues with generalizing results from referential communication tasks to spontaneous conversation.

In conclusion, similar patterns of clarification request were observed in both the pairs consisting of younger adults without hearing impairment, and those consisting of an older adult who was hard of hearing and a younger adult without hearing impairment. An effect of age/hearing impairment on requests for clarification was observed only in the second trial of the referential communication task, with older participants who were hard of hearing making significantly more requests than their partners without hearing impairment. This suggests that fatigue may be an important consideration when planning studies of interaction that use multiple conditions of a communication task, particularly when participants are older or hard of hearing (given the design of the present study, it is not possible to parse out the relative contribution of these two factors). This may also be of relevance if interaction tasks are included towards the end of a test session including other demanding tasks, such as those designed to measure cognitive skills. In addition, the results have important implications for generalizing findings from tasks in which participants have been assigned roles, as only one pair out of 21 in this study naturally chose the describer–receiver dynamic often prescribed when referential communication tasks are used. Furthermore, the findings suggest that patterns of interaction observed in referential communication tasks differ to those observed in more natural interaction, and that the strategy of monopolizing conversation, possibly as a strategy to control it, may be more frequently used by older adults who are hard of hearing in spontaneous conversation than in a more contrived communication task.

## CONFLICT OF INTEREST

The authors report no conflicts of interest.
